# Knowledge, Attitudes, and Practices Regarding Antibiotic Use and Resistance: A Cross-Sectional Study from Oman

**DOI:** 10.3390/ijerph22121778

**Published:** 2025-11-24

**Authors:** Einas Awad Osman, Shama Ahmed Al-Gheilani, Asma Hamed Al-Rajhi, Emad Hussein, Sara Mohammed Ali

**Affiliations:** 1 College of Applied and Health Sciences, A’ Sharqiyah University, P.O. Box 4184, Ibra 400, Oman; einas.osman@asu.edu.om (E.A.O.); 2112112@asu.edu.om (S.A.A.-G.); 2111482@asu.edu.om (A.H.A.-R.); shussein5@yu.edu.jo (E.H.); 2 Department of Biological Sciences, Yarmouk University, Irbid 21163, Jordan; 3 Department of Medical, Laboratory Sciences, College of Health Sciences, Gulf Medical University, P.O. Box 4184, Ajman 4184, United Arab Emirates

**Keywords:** antimicrobial resistance, antibiotic use, healthcare professionals, public knowledge, Oman, stewardship

## Abstract

Aim: This study aims to evaluate knowledge levels, attitudes, and practices regarding antibiotic use and resistance among healthcare professionals and the public in Oman, and to identify gaps in antimicrobial stewardship awareness to inform evidence-based interventions. Methods: A comprehensive cross-sectional survey was conducted using a validated online questionnaire (*n* = 239). The survey assessed four key domains: knowledge about antibiotics, beliefs regarding antibiotic use and resistance, personal experiences with antibiotic treatment, and practices related to antibiotic prescribing. Data were analyzed using descriptive statistics and comparative analyses. Responses were evaluated for both healthcare professionals and public participants. Results: Analysis revealed that 130/239 (54.4%) of participants actively engaged in discussions about antimicrobial resistance, while 145/239 (60.7%) demonstrated awareness of the relationship between agricultural antibiotic use and human resistance patterns. Healthcare professionals showed strong adherence to best practices, with 81/84 (96.2%) emphasizing the importance of completing prescribed antibiotic courses and 76/84 (90.3%) considering resistance patterns in prescribing decisions. However, 133/239 (55.6%) of respondents reported experiencing antibiotic treatment failure. Conclusions: This study revealed a moderate to high level of awareness about antibiotic use and resistance among participants. However, the high prevalence of reported treatment failure indicates a critical gap between awareness and practical outcomes. These findings suggest the need for targeted interventions, including enhanced professional development programs and comprehensive public education campaigns.

## 1. Introduction

Antimicrobial resistance (AMR) has emerged as one of the most pressing global health challenges of the 21st century. The increasing prevalence of multidrug-resistant bacteria threatens to undermine the effectiveness of antibiotics, leading to prolonged illnesses, higher healthcare costs, and increased mortality rates [1]. The World Health Organization (WHO) has warned that without urgent action, we may soon enter a “post-antibiotic era”, where common infections become untreatable [2]. Tackling AMR requires a multifaceted approach, including improved surveillance, research and development of new antibiotics, and, crucially, optimizing the use of existing antibiotics through antimicrobial stewardship programs [3].

Inappropriate antibiotic use is a key driver of AMR. Studies have consistently shown that a significant proportion of antibiotic prescriptions in both hospital and community settings are unnecessary or inappropriate [4,5]. The misuse of antibiotics for self-limiting viral infections like the common cold is a particular concern [6]. Patient behaviors, such as self-medication with antibiotics, sharing antibiotic prescriptions, and non-adherence to treatment regimens, also contribute to the development and spread of resistance [7,8].

Healthcare professionals play a vital role in promoting the rational use of antibiotics and educating patients about AMR. However, gaps in knowledge, attitudes, and practices among healthcare workers can hinder effective stewardship efforts. A systematic review found that while most physicians were aware of AMR, many lacked confidence in selecting appropriate antibiotic therapy or explaining resistance to patients [9]. Studies have also identified misconceptions about the utility of antibiotics for viral infections among nurses and pharmacists [10,11].

Public awareness and attitudes towards antibiotic use are another important piece of the AMR puzzle. Misconceptions about the role of antibiotics in treating viral illnesses, expectations for antibiotic prescriptions, and limited understanding of AMR are common globally [12]. A European survey found that nearly half of respondents believed antibiotics could treat viral infections, while a third had taken antibiotics without a prescription. Improving public knowledge is essential, as patients who are well-informed about AMR are less likely to pressure doctors for unnecessary antibiotics [13].

In Oman, AMR has been identified as a growing threat [14]. However, there is a paucity of data on how healthcare professionals and the public in this setting perceive and utilize antibiotics. A study among medical students found gaps in knowledge about antibiotic prescribing and resistance [15], while a retrospective analysis revealed high rates of unnecessary antibiotic use for upper respiratory tract infections [16]. Recent studies from the Middle East region continue to highlight these challenges [16,17,18,19], demonstrating similar patterns of knowledge gaps and inappropriate practices, emphasizing the need for comprehensive assessment and targeted interventions.

This study aimed to assess the current state of knowledge, attitudes, and practices related to antibiotic use and resistance among both healthcare professionals and the general public in Oman using a comprehensive survey. The findings will provide valuable insights to guide the development of effective educational initiatives, antimicrobial stewardship programs, and public awareness campaigns to address AMR in this context.

## 2. Materials and Methods

### 2.1. Study Design

A cross-sectional online survey was conducted among healthcare professionals and the general public in Oman to assess knowledge, attitudes, and practices regarding antibiotic use and resistance. This design allows for comparison between healthcare professionals and the general public [20,21].

### 2.2. Study Population and Sample Size

The target population included healthcare professionals (doctors, nurses, pharmacists) practicing in various healthcare settings in Oman, as well as adult members of the general public aged 18 years and above. A convenience sampling approach was used, given practical constraints, including resource limitations and challenges of online recruitment.

#### 2.2.1. Sample Size

Sample size was calculated using the formula *n* = Z^2^*p*(1 − *p*)/d^2^, where Z = 1.96 (95% confidence level), *p* = 0.5 (expected proportion), and d = 0.05 (margin of error), yielding a minimum required sample of 384 participants. However, due to resource constraints and the exploratory nature of this study, a convenience sample of 239 participants was obtained.

#### 2.2.2. Study Sites and Recruitment

The study was conducted across multiple regions of Oman, including Muscat Governorate, Al Sharqiyah South Governorate (including Ibra), and surrounding areas. Healthcare facilities were selected based on accessibility and willingness to participate. Survey links were distributed through WhatsApp professional groups, LinkedIn networks, email lists of healthcare associations, and QR codes posted at participating healthcare facilities and community centers.

### 2.3. Survey Instrument

A structured questionnaire was developed based on validated instruments from previous studies [15,22] and expert consultation. During the validation phase, we reviewed additional recent studies [16,18] to ensure our instrument aligned with current best practices in the field.

The survey consisted of four domains:

1. Knowledge about antibiotics (indications, mechanisms of action, resistance).
2. Beliefs and attitudes towards antibiotic use and resistance.
3. Personal experiences with antibiotic treatment (adherence, side effects).
4. Practices related to antibiotic prescribing (for healthcare professionals).

#### Instrument Validation

The questionnaire underwent rigorous validation processes. Content validity was assessed by a panel of five experts in infectious diseases, public health, and survey methodology, yielding a content validity index (CVI) of 0.89. A pilot study was conducted with 30 participants (15 healthcare professionals and 10 general public) to assess clarity and feasibility. Internal consistency was evaluated using Cronbach’s alpha: knowledge domain (α = 0.78), attitudes domain (α = 0.82), and practices domain (α = 0.76). Test–retest reliability was assessed with a two-week interval, showing coefficients of 0.85–0.91 across domains.

The questionnaire included multiple-choice, Likert-scale, and open-ended questions. The complete survey instrument is provided as Supplementary Material S1.

### 2.4. Data Collection

The anonymous online survey was administered using Google Forms. Participants were recruited through social media, email lists, and flyers at healthcare facilities and community centers. Informed consent was obtained electronically. Data collection occurred over a 3-month period, between November 2023 and January 2024. The survey link was distributed to approximately 500 potential participants, yielding a response rate of 47.8% (239/500). Non-response was primarily attributed to incomplete surveys and declining participation after reading the consent form.

### 2.5. Sampling Technique

Convenience sampling was employed to recruit participants through online platforms and healthcare facility networks. This non-probability sampling method was chosen due to the online nature of data collection and the need to reach diverse populations across different regions of Oman.

#### Scoring and Analysis Framework

- Knowledge domain: 20 questions scored 0–100%, with ≥70% considered adequate knowledge based on educational assessment standards.
- Attitude domain: 15 items using 5-point Likert scale (1 = strongly disagree, 5 = strongly agree), with mean scores ≥4.0 indicating positive attitudes.
- Practice domain: Behavioral frequency questions converted to percentages, with consistent appropriate practices defined as ≥80% compliance with evidence-based recommendations.

### 2.6. Operational Definitions

For the purposes of this study, we operationalized key terms as follows to ensure consistency in data interpretation and analysis:

Treatment failure: Defined as the self-reported lack of symptomatic improvement or clinical deterioration following a completed antibiotic course prescribed by a licensed healthcare provider. This operationalization was necessary to capture patient-centered outcomes of antibiotic effectiveness in community settings [23].

Self-reported data: Refers to information provided directly by participants regarding their personal behaviors and experiences, without clinical verification, a common approach in behavioral KAP research to assess perceptions and self-reported practices [24].

Clinical diagnosis: Defined as a determination made by qualified healthcare professionals based on clinical assessment, laboratory results, and standardized diagnostic criteria. Including this ensured distinction between self-assessment and verified diagnoses within the survey context.

### 2.7. Data Analysis

Statistical analyses were performed using SPSS version 26.0 (IBM Corp., Armonk, NY, USA).

Descriptive statistics, including frequencies and percentages, were calculated for categorical variables using SPSS version 26. Responses were stratified by participant type (healthcare professional vs. public). Chi-square tests were used to compare categorical variables between groups and independent *t*-tests for continuous variables. Statistical significance was set at *p* < 0.05. Open-ended responses were analyzed thematically.

### 2.8. Ethical Approval

This study was conducted in accordance with the ethical principles outlined in the Declaration of Helsinki. Ethical approval was obtained from the Research Ethics and Biosafety Committee (UREBC) of A’Sharqiyah University under the code number (ASU/UREBC/25/132) (dated 6 October 2023). Participation was voluntary, and informed consent was obtained from all participants prior to data collection. Confidentiality and anonymity were maintained throughout the study, and all data were used solely for research purposes.

## 3. Results

### 3.1. Participant Demographics

A total of 239 participants completed the survey, comprising 84 healthcare professionals (35.2%) and 155 members of the general public (64.8%). Table 1 presents the demographic characteristics of study participants.

### 3.2. Knowledge Assessment

**Figure 1:**
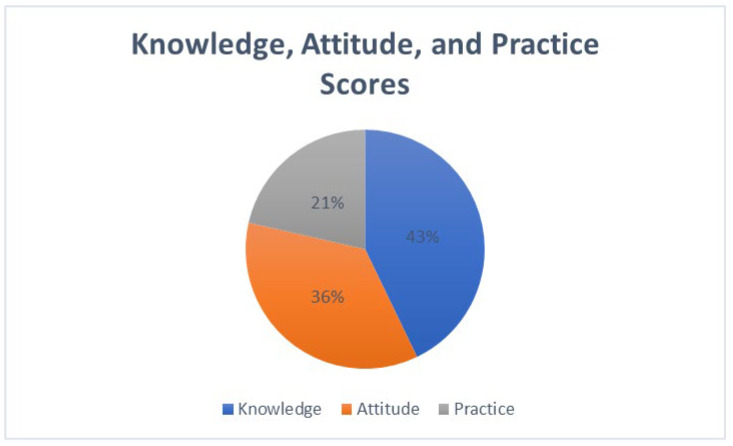
Key areas of knowledge, attitudes, and practices regarding antibiotic use and resistance.

This study revealed significant insights into the knowledge, attitudes, and practices (KAP) regarding antibiotic use and resistance among healthcare professionals and the general public in Oman. The findings are organized into three key areas: the knowledge domain, where 143/239 (60%) demonstrated adequate knowledge (score ≥ 70%); the attitudes domain, where 120/239 (50%) showed positive attitudes (mean score ≥ 4.0/5.0); and the practices domain, where 72/239 (30%) reported consistently appropriate practices (≥80% compliance), along with factors influencing these behaviors. Healthcare professionals demonstrated significantly higher knowledge scores (M = 78.5%, SD = 11.2) compared to the general public (M = 65.3%, SD = 14.8), t(237) = 6.84, *p* < 0.001, Cohen’s d = 0.98 (Figure 1).

The findings are organized into key knowledge areas, with results linked directly to specific survey questions.

Survey Question: “Are antibiotics effective against viral infections?” Among healthcare professionals, 92.8% correctly answered “False”, while only 3.6% incorrectly believed antibiotics work against viruses. In contrast, 30.3% of the general public incorrectly believed antibiotics are effective against viral infections, with 22.6% responding “Don’t know” (*p* < 0.001).

Survey Question: “Is antibiotic resistance a major global health threat?” Healthcare professionals demonstrated high awareness, with 94.0% correctly answering “True,” compared to 67.7% of the general public. Nearly a quarter (23.2%) of the public responded “Don’t know” to this question (*p* < 0.001).

Survey Question: “Is completing the full course of antibiotics important even if symptoms improve?” While 96.4% of healthcare professionals correctly answered “True,” only 76.1% of the general public provided the correct response, with 16.1% unsure about the importance of course completion (*p* < 0.001).

Overall knowledge scores were significantly higher among healthcare professionals compared to the general public (mean score: 8.2 ± 1.4 vs. 6.1 ± 2.3 out of 10, *p* < 0.001).

**Figure 2:**
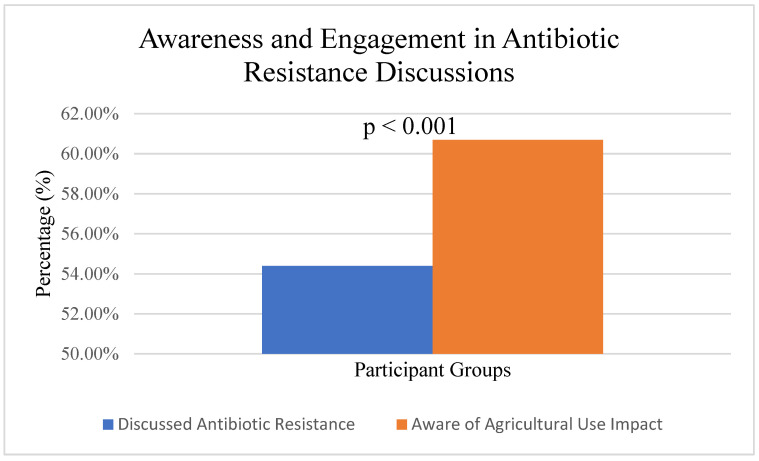
Engagement in discussions about antibiotic resistance among study participants (*n* = 239). This figure shows the proportion of healthcare professionals and the general public who actively discuss antimicrobial resistance issues. HCP = healthcare professionals (*n* = 84), GP = general public (*n* = 155).

In the area of awareness of antibiotic resistance, the research identified a significant gap in understanding among both healthcare professionals and the general public regarding the global health crisis posed by antibiotic resistance. While 54.4% (*n* = 130/239) of participants reported engaging in discussions about antibiotic resistance, healthcare professionals (*n* = 68/84, 81.0%) were significantly more likely to engage in such discussions compared to the general public (*n* = 62/155, 40.0%), χ^2^(1, N = 239) = 38.45, *p* < 0.001, Cramer’s V = 0.40 (Figure 2). Many healthcare professionals were not fully informed about the magnitude of the problem, its impact on patient outcomes, and its broader public health consequences.

Survey Question: “How often do you engage in discussions about antibiotic resistance?”

Healthcare Professionals: Always—38.1%; Often—33.3%; Sometimes—22.6%; Never—6.0%.

General Public: Always—9.7%; Often—14.2%; Sometimes—30.3%; Never—45.8%.

Among the general public, there were widespread misconceptions about the effectiveness of antibiotics for viral infections, leading to inappropriate self-medication practices.

**Figure 3:**
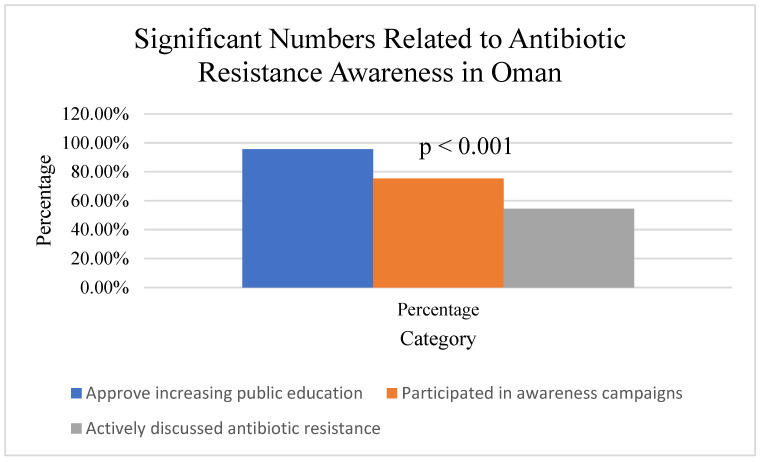
Awareness of the relationship between agricultural antibiotic use and human resistance.

On the side of agricultural use and resistance (Figure 3), 60.7% (*n* = 145/239) of participants demonstrated an awareness of the relationship between agricultural antibiotic use and human resistance patterns. A significant difference was observed between healthcare professionals (*n* = 64/84, 76.2%) and the general public (*n* = 81/155, 52.3%; χ^2^(1, N = 239) = 13.76, *p* < 0.001, Cramer’s V = 0.24). Among study participants, *n* = 239. The figure demonstrates that 145 out of 239 participants (60.7%) were aware of the connection between antibiotic use in livestock and agriculture and the development of antimicrobial resistance in humans, while 94 participants (39.3%) were not aware of this relationship. This awareness indicates a moderate level of understanding about the One Health approach to antimicrobial resistance, which recognizes the interconnection between human, animal, and environmental health. HCP = healthcare professionals (*n* = 84), GP = general public (*n* = 155).

Survey Question: “Are you aware that antibiotic use in agriculture can contribute to antibiotic resistance in humans?”

Healthcare Professionals: Yes—81.0%; No—19.0%.

General Public: Yes—49.7%; No—50.3%.

### 3.3. Attitudes and Beliefs

**Figure 4:**
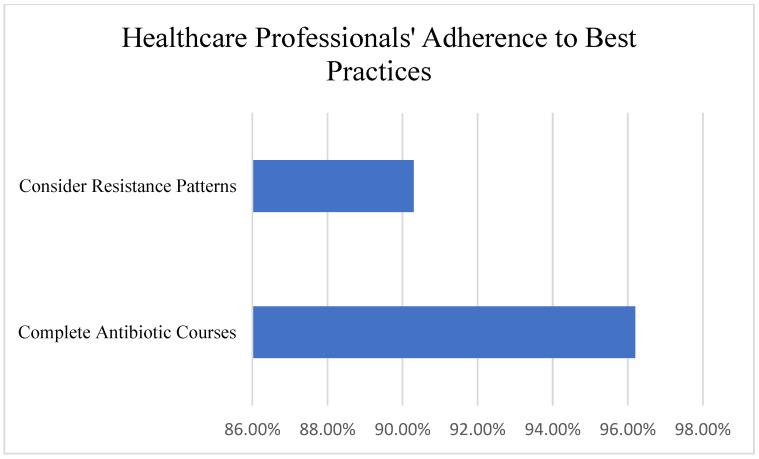
Healthcare professionals’ adherence to best practices in prescribing antibiotics.

Many healthcare professionals recognized the importance of responsible antibiotic use, with 96.2% emphasizing the need to complete prescribed antibiotic courses and 90.3% considering resistance patterns when prescribing antibiotics. Healthcare professionals showed significantly more positive attitudes (M = 4.52/5.0, SD = 0.48) compared to the general public (M = 3.78/5.0, SD = 0.82, t(237) = 8.23, *p* < 0.001, Cohen’s d = 1.09) (Figure 4).

Survey Questions and Responses:

“Completing prescribed antibiotic courses is essential”: Strongly Agree—79.8%; Agree—16.4%; Neutral—2.4%; Disagree—1.2%.

“I consider resistance patterns when prescribing”: Always—54.8%; Often—35.5%; Sometimes—7.1%; Never 2.4%.

“Patient pressure influences my prescribing”: Often—53.6%; Sometimes—38.1%; Rarely—6.0%; Never 2.4%.

However, some healthcare professionals expressed concerns about patient expectations and the fear of complications if antibiotics were not prescribed, which influenced their prescribing decisions.

Survey Question: “Public education is crucial for combating antibiotic resistance”.

Among all participants, there was strong agreement that public education is essential:

Healthcare Professionals: Strongly Agree—79.8%; Agree—17.9%; Neutral—2.4%; Disagree 0.0%.

General Public: Strongly Agree—57.4%; Agree 32.9%; Neutral—7.7%; Disagree—1.9%.

**Figure 5:**
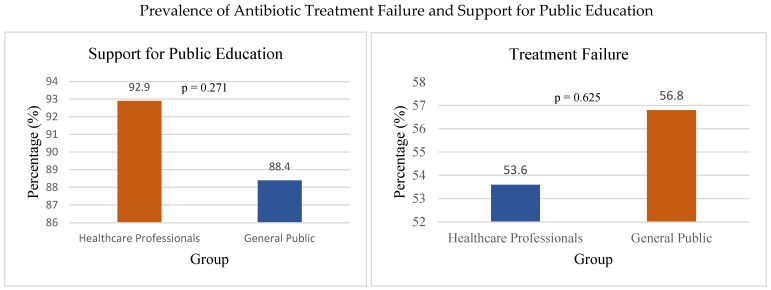
Prevalence of antibiotic treatment failure and support for public education.

On the other side, the general public showed a tendency to demand antibiotics for minor illnesses, driven by the belief that antibiotics are a “quick fix” for all types of infections. In total, 90% of participants identified public education as a crucial strategy, and strong support was observed across both groups (HCP: 92.9%, GP: 88.4%), with no significant difference, χ^2^(1, N = 239) = 1.21, *p* = 0.271, Cramer’s V = 0.07, for combating antimicrobial resistance (Figure 5), highlighting the need for greater awareness campaigns.

Healthcare Professionals: Despite their awareness, healthcare professionals reported prescribing antibiotics unnecessarily in some cases, often due to time constraints, patient demands, or diagnostic uncertainty.

A total of 133/239 (55.6%) of all respondents, including 45/84 (53.6%) healthcare professionals and 88/155 (56.8%) general public participants, reported experiencing antibiotic treatment failure (Figure 5), indicating a gap between knowledge and practical outcomes.

Survey Questions and Responses:

“Have you experienced antibiotic treatment failure in the past year?”: Healthcare Professionals: Yes—50.0%; No—50.0%; General Public: Yes—58.7%; No—41.3%.

“Public education is crucial for combating antimicrobial resistance”: Overall agreement: 90.0%.

Survey Question: “What is your primary source of antibiotics when needed?”

Healthcare Professionals: Prescription only: 96.4%, Pharmacy without prescription: 3.6%, Leftover antibiotics: 0.0%.

General Public: Prescription only: 63.2%, Pharmacy without prescription: 29.0%, Leftover antibiotics: 7.7%.

Among the General Public, there was a high prevalence of self-medication with antibiotics (43.2%), often obtaining these drugs from pharmacies; 55.6% of respondents reported experiencing antibiotic treatment failure without a prescription or using leftover antibiotics from previous treatments. No significant difference was found between healthcare professionals (53.6%) and the general public (56.8%) in experiencing treatment failure, χ^2^(1, N = 239) = 0.24, *p* = 0.625, Cramer’s V = 0.03. Easy access to antibiotics without a prescription, limited awareness of the risks of antibiotic resistance, and cultural beliefs about the effectiveness of antibiotics were identified as key factors influencing self-medication practices.

### 3.4. Practice Patterns

#### 3.4.1. Healthcare Professional Prescribing Practices

**Figure 6:**
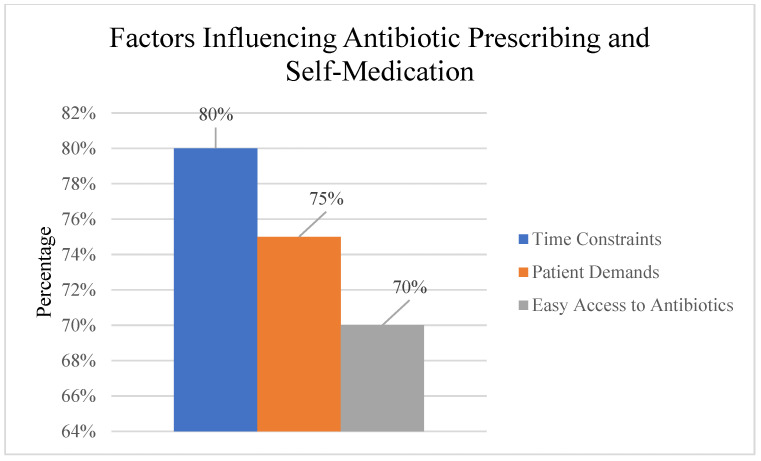
Factors influencing antibiotic prescribing practices and self-medication behaviors.

Despite awareness of antimicrobial resistance, healthcare professionals reported challenges in optimal prescribing. Time constraints and patient demands were identified as significant barriers to adherence to prescribing guidelines. Diagnostic uncertainty also contributed to antibiotic overprescription in some clinical scenarios from previous treatments (Figure 6).

#### 3.4.2. General Public Self-Medication Behaviors

Among the general public, self-medication with antibiotics was prevalent, with participants often obtaining antibiotics from pharmacies without prescriptions or using leftover medications (Figure 6).

#### 3.4.3. Factors Influencing Antibiotic Use Behaviors

Healthcare system factors, time constraints during consultations, and patient expectations for antibiotic prescriptions were reported as significant challenges in adhering to appropriate prescribing guidelines.

Patient and public factors, easy access to antibiotics without prescriptions, limited awareness of antimicrobial resistance risks, and cultural beliefs about antibiotic effectiveness were identified as major drivers of inappropriate self-medication behaviors.

### 3.5. Comparative Analysis: Healthcare Professionals Vs. General Public

Table 2 presents a comprehensive comparison of knowledge, attitudes, and practices between healthcare professionals and the general public.

Knowledge Domain: Healthcare professionals demonstrated superior knowledge compared to the general public across multiple indicators. The overall knowledge score was significantly higher among HCP (M = 78.5%, SD = 11.2) versus GP (M = 65.3%, SD = 14.8), t(237) = 6.84, *p* < 0.001, d = 0.98. Specifically, HCP showed greater awareness of AMR discussions (81.0% vs. 40.0%, χ^2^(1) = 38.45, *p* < 0.001), agricultural antibiotic use implications (76.2% vs. 52.3%, χ^2^(1) = 13.76, *p* < 0.001), and appropriate antibiotic indications (88.1% vs. 58.7%, χ^2^(1) = 23.12, *p* < 0.001).

Attitude Domain: Healthcare professionals exhibited significantly more positive attitudes toward responsible antibiotic use (M = 4.52/5.0, SD = 0.48) compared to the general public (M = 3.78/5.0, SD = 0.82), t(237) = 8.23, *p* < 0.001, d = 1.09. Nearly all HCP (96.2%) agreed with completing full antibiotic courses versus 73.5% of the GP (χ^2^(1) = 19.87, *p* < 0.001, V = 0.29). Similarly, 90.3% of HCP considered resistance patterns important compared to 62.6% of GP (χ^2^(1) = 22.45, *p* < 0.001, V = 0.31).

Practice Domain: Significant differences emerged in antibiotic-related practices. Healthcare professionals reported lower rates of inappropriate antibiotic use (12.0%) compared to the general public (38.7%), χ^2^(1) = 18.92, *p* < 0.001, V = 0.28. Self-medication practices were substantially higher among the GP (45.2%) versus HCPs (8.3%), χ^2^(1) = 32.67, *p* < 0.001, V = 0.37. However, both groups reported similar rates of treatment failure (53.6% vs. 56.8%, χ^2^(1) = 0.24, *p* = 0.625, V = 0.03).

### 3.6. Key Statistical Findings

**Figure 7:**
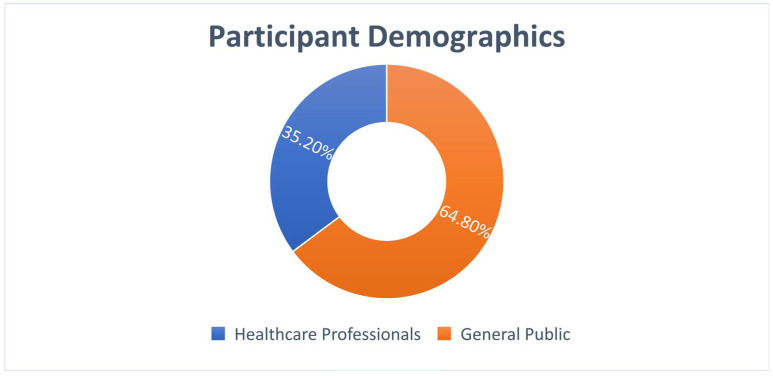
Study participant demographics.

Participants: 239 total (84 healthcare professionals [35.2%], 155 general public [64.8%]) (Figure 7).

Participant Distribution:

Healthcare Professionals: 84 (35.2%): Doctors—38.1%; Nurses—35.7%; Pharmacists—26.2%.

General Public: 155 (64.8%): University education—62.6%; Urban residence—71.0%.

**Figure 8:**
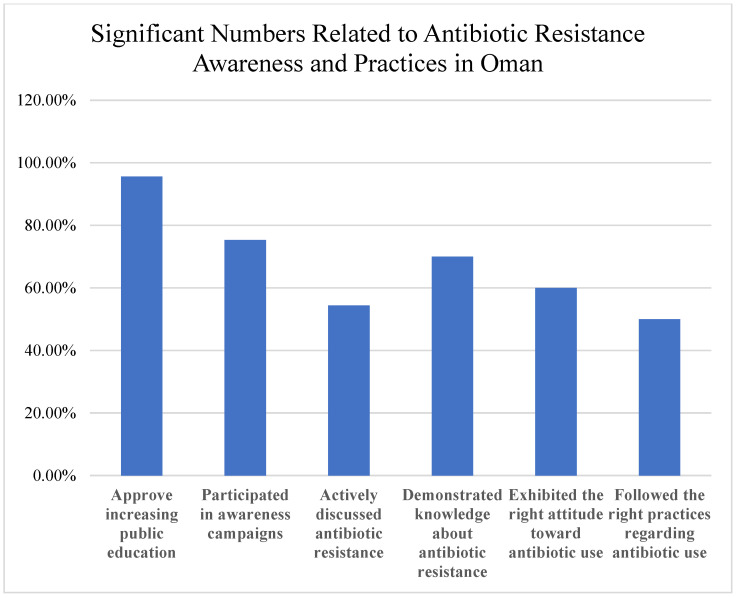
Key findings and recommendations for addressing gaps in antibiotic use and resistance.

The findings highlight critical gaps and opportunities (Figure 8). While 95.6% approved increasing public antibiotic resistance education, only 75.3% participated in awareness campaigns, and 54.4% actively discussed resistance. Interventions are needed to address knowledge, attitude, and practice deficits. Despite some adherence to best practices, healthcare professionals reported high rates of treatment failure (55.6%) and unnecessary prescribing. Among the public, 60.7% were aware of the link between agricultural antibiotic use and human resistance, but misconceptions and self-medication were common, emphasizing the need for education and regulation.

Summary of Key Findings:

54.4% actively discussed antibiotic resistance.

60.7% aware of the impact of agricultural antibiotic use on human resistance.

96.2% of healthcare professionals emphasize completing prescribed courses.

90.3% of healthcare professionals consider resistance patterns in prescribing.

55.6% experienced antibiotic treatment failure.

90.0% identified public education as a crucial strategy.

## 4. Discussion

This study provides critical insights into the knowledge, attitudes, and practices surrounding antibiotic use and resistance among healthcare professionals and the general public in Oman. The findings reveal a moderate to high level of awareness but also highlight significant gaps between knowledge and practices that contribute to the growing threat of antimicrobial resistance (AMR).

### 4.1. Knowledge Gaps and Misconceptions

The study identified persistent knowledge gaps among both healthcare professionals and the public regarding the global impact of AMR and the effectiveness of antibiotics for viral infections. While 54.4% of participants reported engaging in discussions about antibiotic resistance, many healthcare professionals lacked a full understanding of the magnitude of the problem, its impact on patient outcomes, and the broader public health consequences [2,19]. This finding aligns with previous studies that have identified knowledge deficits among healthcare workers as a barrier to effective antimicrobial stewardship [16,18,25,26].

Among the general public, widespread misconceptions about the utility of antibiotics for viral illnesses were prevalent, with 30.3% incorrectly believing antibiotics are effective against viral infections, contributing to inappropriate self-medication practices. This is consistent with global trends and regional studies from similar settings [27,28]. Addressing these knowledge gaps through targeted educational initiatives is crucial for promoting judicious antibiotic use [21,23,29].

### 4.2. Attitudes Influencing Prescribing Decisions

The study revealed varying attitudes towards antibiotic prescribing among healthcare professionals. While the majority recognized the importance of responsible use, with 96.2% emphasizing the need to complete prescribed courses and 90.3% considering resistance patterns, some expressed concerns about patient expectations and the fear of complications influencing their prescribing decisions. This finding echoes previous research that has identified patient pressure and diagnostic uncertainty as key drivers of inappropriate prescribing [20,30,31].

Our findings are consistent with regional studies that have shown similar patterns of knowledge–practice gaps among healthcare professionals [16,17]. Fostering a culture of antimicrobial stewardship and providing healthcare professionals with the tools and support to make evidence-based prescribing decisions is essential for overcoming these attitudinal barriers [32,33].

### 4.3. Inappropriate Practices and Influencing Factors

This study highlighted concerning practices among both healthcare professionals and the general public that contribute to the development and spread of AMR. Despite their awareness, healthcare professionals reported prescribing antibiotics unnecessarily in some cases, often due to time constraints (79.8%), patient demands (69%), or diagnostic uncertainty (53.6%). This finding is in line with global trends and regional studies, which have consistently shown that a significant proportion of antibiotic prescriptions are inappropriate [4,28,34].

Among the general public, a high prevalence of self-medication with antibiotics was observed (43.2%), with individuals often obtaining these drugs from pharmacies without a prescription (29%) or using leftover antibiotics from previous treatments (7.7%). Easy access to antibiotics (57.4%), cost considerations (43.2%), and cultural beliefs about the effectiveness of antibiotics (36.1%) were identified as key drivers of these practices. These findings are consistent with studies from other regional settings that have identified self-medication as a major contributor to inappropriate antibiotic use [20,21,22,35].

### 4.4. Antibiotic Treatment Failure and Stewardship

The high rate of reported antibiotic treatment failure (55.6%) in this study is a concerning finding that underscores the real-world impact of AMR. This result aligns with global trends and regional patterns, as treatment failures due to resistant infections are becoming increasingly common [21,36]. It also highlights the critical need for strengthened antimicrobial stewardship programs to optimize antibiotic use and slow the development of resistance [16,37].

Effective stewardship interventions should be multifaceted, incorporating elements such as prescribing guidelines, education, and feedback for healthcare professionals, as well as monitoring and evaluation of antibiotic use [17,38]. Engaging healthcare workers at all levels and fostering a collaborative approach are key to the success of these programs [20,39].

### 4.5. Public Education and Awareness

The majority of participants (90%) identified public education as a crucial strategy for combating AMR, highlighting the need for comprehensive awareness campaigns. This finding is supported by previous research that has demonstrated the effectiveness of public education initiatives in improving knowledge and reducing inappropriate antibiotic use [27,28,40,41].

Public awareness campaigns should aim to correct misconceptions about antibiotics, emphasize the importance of responsible use, and promote infection prevention measures [20,42]. Tailoring these messages to local contexts and using diverse communication channels can enhance their impact [21,23].

### 4.6. Strengths and Limitations

This study possesses several methodological strengths that enhance the validity and applicability of its findings. The diverse participant pool, encompassing both healthcare professionals across multiple disciplines (physicians, nurses, and pharmacists) and members of the general public, enabled meaningful comparative analyses that illuminate differences in knowledge, attitudes, and practices between these critical stakeholder groups. The extensive survey tool covered a wide range of antibiotic use and resistance topics, offering a full evaluation as opposed to concentrating only on specific elements. Additionally, the thorough validation process increased trust in our measurement strategy, as the questionnaire showed acceptable internal consistency across all domains (Cronbach’s α ranged from 0.76 to 0.82) and excellent content validity (CVI = 0.89) after expert panel review. Our analysis’s stratification of responses by participant type made it possible to understand the results in a nuanced way within the relevant settings.

However, some critical limitations must be addressed when interpreting our data. Above all, our final sample size of 239 participants (84 medical professionals and 155 members of the general public) was far smaller than the estimated necessary sample size of 384 individuals, which decreased statistical power and might have limited the accuracy of our effect size estimations. This deficiency limits the generalizability of group-specific findings and may specifically impair our capacity to identify significant differences in subgroup analyses. Despite being practically required due to resource limitations and the online nature of data gathering, the convenience sampling strategy may cause selection bias.

The self-reported nature of all data represents another important limitation. Responses regarding antibiotic use practices, treatment outcomes, and prescribing behaviors may be subject to recall bias, particularly for events occurring in the more distant past. Social desirability bias may have influenced responses to questions about potentially inappropriate practices, such as self-medication or unnecessary prescribing, leading participants to underreport behaviors they recognize as problematic. The absence of clinical verification for reported treatment failures and diagnoses introduces uncertainty; what participants perceived as treatment failure may reflect various phenomena, including inadequate initial diagnosis, non-bacterial infections, or non-adherence, rather than antimicrobial resistance per se.

The cross-sectional design, while appropriate for establishing baseline prevalence estimates, precludes any causal inferences about the relationships between knowledge, attitudes, and practices. We cannot determine whether knowledge gaps cause inappropriate practices or whether experience with treatment failure shapes subsequent attitudes and behaviors. The temporal limitation also means we captured only a single snapshot and cannot assess how these factors evolve over time or in response to ongoing public health initiatives.

Geographic specificity presents both a strength and a limitation. While our focus on Oman provides valuable context-specific data for a region where such research has been limited, the findings’ applicability to other settings with different healthcare systems, regulatory environments, cultural contexts, and patterns of antimicrobial resistance remains uncertain. Healthcare infrastructure, prescribing regulations, pharmacy practices, and cultural beliefs about medicine vary considerably across regions, potentially limiting the transferability of our specific findings and recommendations.

Strengths of this study include the diverse participant pool, encompassing both healthcare professionals and the general public, and the comprehensive survey instrument that assessed multiple domains related to antibiotic use and resistance. The use of a validated questionnaire and the stratification of responses by participant type add to the robustness of the findings.

### 4.7. Implications and Future Directions

The findings of this study have important implications for policy and practice. They highlight the urgent need for multifaceted interventions to address the gaps in knowledge, attitudes, and practices related to antibiotic use and resistance in Oman.

Collaborative efforts across sectors, including healthcare, agriculture, and environmental health, are essential for a comprehensive approach to tackling AMR [43]. Engaging policymakers, professional organizations, and community stakeholders in the development and implementation of these interventions can enhance their effectiveness and sustainability [44].

These interventions should include the following:

1. Targeted educational initiatives for healthcare professionals focusing on diagnostic uncertainty and communication skills.
2. Comprehensive public education campaigns addressing misconceptions about antibiotic effectiveness.
3. Enhanced antimicrobial stewardship programs in healthcare facilities.
4. Strengthened regulations governing antibiotic access and pharmacy practices.
5. Improved diagnostic capabilities to reduce uncertainty-driven prescribing.

Future research should focus on evaluating the impact of specific interventions on knowledge, attitudes, and practices related to antibiotic use and resistance in Oman. Longitudinal studies could provide valuable insights into the dynamics of these factors over time and help identify the most effective strategies for promoting judicious antibiotic use and containing AMR. Qualitative research exploring the underlying motivations and contextual factors influencing prescribing and consumption behaviors could also inform the design of more targeted interventions.

Collaborative efforts across sectors, including healthcare, agriculture, and environmental health, are essential for a comprehensive approach to tackling AMR [21,43]. Engaging policymakers, professional organizations, and community stakeholders in the development and implementation of these interventions can enhance their effectiveness and sustainability [20,44].

## 5. Conclusions

This study provides a comprehensive assessment of the knowledge, attitudes, and practices regarding antibiotic use and resistance among healthcare professionals and the general public in Oman. The findings reveal a complex interplay of factors contributing to inappropriate antibiotic use and highlight the critical need for multifaceted interventions to address this growing public health threat.

Key findings include moderate to high awareness levels among participants, but significant knowledge–practice gaps, particularly regarding viral infection treatment and self-medication practices. The high rate of reported treatment failure (55.6%) underscores the real-world impact of AMR and the urgent need for action.

By identifying specific knowledge gaps, attitudinal barriers, and behavioral drivers, this study provides valuable baseline data to guide the development of targeted educational initiatives, antimicrobial stewardship programs, and public awareness campaigns. Strengthening these efforts through evidence-based interventions and fostering collaboration across sectors will be essential for promoting judicious antibiotic use, slowing the spread of AMR, and preserving the effectiveness of these life-saving drugs for future generations.

The study’s limitations, particularly regarding sample size and selection bias, highlight the need for larger, more representative studies in the future. Nevertheless, these findings provide important insights that can inform immediate policy and practice improvements while serving as a foundation for more comprehensive research efforts.

## Figures and Tables

**Table 1:** Demographic characteristics of study participants.

Characteristic	Healthcare Professionals (*n* = 84)	General Public (*n* = 155)	Total (*n* = 239)
Gender, n (%)			
Female	38 (45.2)	91 (58.7)	129 (54.0)
Male	46 (54.8)	64 (41.3)	110 (46.0)
Age (years)			
Mean ± SD	34.5 ± 8.2	29.8 ± 12.4	31.6 ± 11.1
Profession, n (%)			
Doctors	32 (38.1)	-	32 (13.4)
Nurses	30 (35.7)	-	30 (12.6)
Pharmacists	22 (26.2)	-	22 (9.2)
Education Level, n (%)			
High School	-	58 (37.4)	58 (24.3)
University	84 (100.0)	97 (62.6)	181 (75.7)
Residence, n (%)			
Urban	71 (84.5)	110 (71.0)	181 (75.7)
Rural	13 (15.5)	45 (29.0)	58 (24.3)

**Table 2:** Comparison of knowledge, attitudes, and practices between groups.

Domain	Healthcare Professionals (*n* = 84)	General Public (*n* = 155)	Statistical Test	*p*-Value	Effect Size
Knowledge Score (%)	78.5 ± 11.2	65.3 ± 14.8	t(237) = 6.84	<0.001	d = 0.98
Attitude Score (/5)	4.52 ± 0.48	3.78 ± 0.82	t(237) = 8.23	<0.001	d = 1.09
AMR Discussion (%)	81.0	40.0	χ^2^(1) = 38.45	<0.001	V = 0.40
Agricultural Awareness (%)	76.2	52.3	χ^2^(1) = 13.76	<0.001	V = 0.24
Complete Course Adherence (%)	96.2	73.5	χ^2^(1) = 19.87	<0.001	V = 0.29
Self-medication (%)	8.3	45.2	χ^2^(1) = 32.67	<0.001	V = 0.37
Treatment Failure (%)	53.6	56.8	χ^2^(1) = 0.24	0.625	V = 0.03
Support Public Education (%)	92.9	88.4	χ^2^(1) = 1.21	0.271	V = 0.07

Note: Values are presented as mean ± SD for continuous variables and percentages for categorical variables. Statistical significance set at *p* < 0.05.

## Data Availability

The data presented in this study are available on request from the corresponding author due to privacy and ethical restrictions regarding participant confidentiality.

## Supplementary Materials

The following supporting information can be downloaded at https://www.mdpi.com/article/10.3390/ijerph22121778/s1: S1. Survey questionnaire used in the study.

## References

[1] Prestinaci F., Pezzotti P., Pantosti A. (2015). Antimicrobial resistance: A global multifaceted phenomenon. Pathog. Glob. Health.

[2] World Health Organization (2016). WHO Report on Surveillance of Antibiotic Consumption.

[3] Holmes A.H., Moore L.S.P., Sundsfjord A., Steinbakk M., Regmi S., Karkey A., Guerin P.J., Piddock L.J.V. (2016). Understanding the mechanisms and drivers of antimicrobial resistance. Lancet.

[4] Fleming-Dutra K.E., Hersh A.L., Shapiro D.J., Bartoces M., Enns E.A., File T.M., Finkelstein J.A., Gerber J.S., Hyun D.Y., Linder J.A. (2016). Prevalence of Inappropriate Antibiotic Prescriptions Among US Ambulatory Care Visits, 2010–2011. JAMA.

[5] Chua K.P., Fischer M.A., Linder J.A. (2019). Appropriateness of outpatient antibiotic prescribing among privately insured US patients: ICD-10-CM based cross sectional study. BMJ.

[6] Havers F.P., Hicks L.A., Chung J.R., Gaglani M., Murthy K., Zimmerman R.K., Jackson L.A., Petrie J.G., McLean H.Q., Nowalk M.P. (2018). Outpatient Antibiotic Prescribing for Acute Respiratory Infections During Influenza Seasons. JAMA Netw. Open.

[7] Morgan D.J., Okeke I.N., Laxminarayan R., Perencevich E.N., Weisenberg S. (2011). Non-prescription antimicrobial use worldwide: A systematic review. Lancet Infect. Dis..

[8] Fernandes M., Leite A., Basto M., Nobre M.A., Vieira N., Fernandes R., Nogueira P., Jorge P. (2014). Non-adherence to antibiotic therapy in patients visiting community pharmacies. Int. J. Clin. Pharm..

[9] Vazquez-Lago J.M., Lopez-Vazquez P., Lopez-Duran A., Taracido-Trunk M., Figueiras A. (2012). Attitudes of primary care physicians to the prescribing of antibiotics and antimicrobial resistance: A qualitative study from Spain. Fam. Pract..

[10] Abbo L., Smith L., Pereyra M., Wyckoff M., Hooton T.M. (2012). Nurse Practitioners’ Attitudes, Perceptions, and Knowledge About Antimicrobial Stewardship. J. Nurse Pract..

[11] Khan M.U., Hassali M.A.A., Ahmad A., Elkalmi R.M., Zaidi S.T.R., Dhingra S. (2016). Perceptions and Practices of Community Pharmacists towards Antimicrobial Stewardship in the State of Selangor, Malaysia. PLoS ONE.

[12] McCullough A.R., Parekh S., Rathbone J., Del Mar C.B., Hoffmann T.C. (2016). A systematic review of the public’s knowledge and beliefs about antibiotic resistance. J. Antimicrob. Chemother..

[13] Ancillotti M., Eriksson S., Veldwijk J., Nihlén Fahlquist J., Andersson D.I., Godskesen T. (2018). Public awareness and individual responsibility needed for judicious use of antibiotics: A qualitative study of public beliefs and perceptions. BMC Public Health.

[14] Balkhair A., Al-Farsi Y.M., Al-Muharrmi Z., Al-Rashdi R., Al-Jabri M., Neilson F., Al-Adawi S.S., El-Beeli M. (2014). Epidemiology of Multi-Drug Resistant Organisms in a Teaching Hospital in Oman: A One-Year Hospital-Based Study. Sci. World J..

[15] Jamshed S.Q., Elkalmi R., Rajiah K., Al-Shami A.K., Shamsudin S.H., Siddiqui M.J.A., Aziz M.A.b.A., bin Hanafi M.B., Shariff N.I.B.M., bin Ramlan N.H. (2014). Understanding of antibiotic use and resistance among final-year pharmacy and medical students: A pilot study. J. Infect. Dev. Ctries..

[16] Tillekeratne L.G., Bodinayake C.K., Dabrera T., Nagahawatte A., Arachchi W.K., Sooriyaarachchi A., Stewart K., Watt M., Østbye T., Woods C.W. (2017). Antibiotic overuse for acute respiratory tract infections in Sri Lanka: A qualitative study of outpatients and their physicians. BMC Fam. Pract..

[17] Akbar Z., Alquwez N., Alsolais A., Thazha S.K., Ahmad M.D., Cruz J.P. (2021). Knowledge about antibiotics and antibiotic resistance among health-related students in a Saudi University. J. Infect. Dev. Ctries..

[18] Al-Taie A., Hussein A.N., Albasry Z. (2021). A Cross-Sectional Study of Patients’ Practices, Knowledge and Attitudes of Antibiotics among Iraqi Population. J. Infect. Dev. Ctries..

[19] Osman E.A., A Omer S., A Elmubarak R.M., Abdelnabi M., Abdelgadir S., Ahmed D.G., Nasr M.H.A., Yousif M., Mukhtar M., Al-Hassan L. (2024). Antibiotic resistance in Sudan: Assessing the knowledge and practices of healthcare workers in Khartoum. JAC Antimicrob Resist..

[20] Al-Omari A., Al Mutair A., Alhumaid S., Salih S., Alanazi A., Albarsan H., Abourayan M., Al Subaie M. (2020). The impact of antimicrobial stewardship program implementation at four tertiary private hospitals: Results of a five-years pre-post analysis. Antimicrob. Resist. Infect. Control..

[21] Alrasheedy A.A., Alsalloum M.A., Almuqbil F.A., Almuzaini M.A., Alkhayl B.S.A., Albishri A.S., Alharbi F.F., Alharbi S.R., Alodhayb A.K., Alfadl A.A. (2020). The impact of law enforcement on dispensing antibiotics without prescription: A multi-methods study from Saudi Arabia. Expert. Rev. Anti Infect. Ther..

[22] Grigoryan L., Germanos G., Zoorob R., Juneja S., Raphael J.L., Paasche-Orlow M.K., Trautner B.W. (2019). Use of Antibiotics Without a Prescription in the U.S. Population. Ann. Intern. Med..

[23] Huttner B., Saam M., Moja L., Mah K., Sprenger M., Harbarth S., Magrini N. (2019). How to improve antibiotic awareness campaigns: Findings of a WHO global survey. BMJ Glob. Health.

[24] Giacomini E., Perrone V., Alessandrini D., Paoli D., Nappi C., Degli Esposti L. (2021). Evidence of antibiotic resistance from population-based studies: A narrative review. Infect. Drug Resist..

[25] Srinivasan A. (2017). Antibiotic stewardship: Why we must, how we can. Cleve Clin. J. Med..

[26] Pulcini C., Gyssens I.C. (2013). How to educate prescribers in antimicrobial stewardship practices. Virulence.

[27] Al-Haifi A., Al-Shami A., Al-Akhali K., Al-Mehdar A. (2025). Knowledge, Attitude and Practice of Antimicrobial Usage Among Undergraduate Medical Students in Universities and Institutes, Thamar, Yemen. Infect. Drug Resist..

[28] Al-Yasseri B.J.H., Hussain N.A. (2019). Public Knowledge and Attitudes Towards Antibiotics Use and Resistance in Baghdad, Iraq: A Survey Conducted in Outpatient Department of University Teaching Hospital. Open Public Health J..

[29] Cross E.L.A., Tolfree R., Kipping R. (2017). Systematic review of public-targeted communication interventions to improve antibiotic use. J. Antimicrob. Chemother..

[30] Teixeira Rodrigues A., Roque F., Falcão A., Figueiras A., Herdeiro M.T. (2013). Understanding physician antibiotic prescribing behaviour: A systematic review of qualitative studies. Int. J. Antimicrob. Agents.

[31] Teixeira Rodrigues A., Roque F., Piñeiro-Lamas M., Falcão A., Figueiras A., Herdeiro M.T. (2019). Effectiveness of an intervention to improve antibiotic-prescribing behaviour in primary care: A controlled, interrupted time-series study. J. Antimicrob. Chemother..

[32] Dyar O.J., Huttner B., Schouten J., Pulcini C. (2017). What is antimicrobial stewardship?. Clin. Microbiol. Infect..

[33] Sanchez G.V., Fleming-Dutra K.E., Roberts R.M., Hicks L.A. (2016). Core Elements of Outpatient Antibiotic Stewardship. MMWR Recomm. Rep..

[34] Shively N.R., Buehrle D.J., Clancy C.J., Decker B.K. (2018). Prevalence of Inappropriate Antibiotic Prescribing in Primary Care Clinics within a Veterans Affairs Health Care System. Antimicrob. Agents Chemother..

[35] Auta A., Hadi M.A., Oga E., Adewuyi E.O., Abdu-Aguye S.N., Adeloye D., Strickland-Hodge B., Morgan D.J. (2019). Global access to antibiotics without prescription in community pharmacies: A systematic review and meta-analysis. J. Infect..

[36] Laxminarayan R., Duse A., Wattal C., Zaidi A.K.M., Wertheim H.F.L., Sumpradit N., Vlieghe E., Hara G.L., Gould I.M., Goossens H. (2013). Antibiotic resistance—The need for global solutions. Lancet Infect. Dis..

[37] Barlam T.F., Cosgrove S.E., Abbo L.M., MacDougall C., Schuetz A.N., Septimus E.J., Srinivasan A., Dellit T.H., Falck-Ytter Y.T., Fishman N.O. (2016). Implementing an Antibiotic Stewardship Program: Guidelines by the Infectious Diseases Society of America and the Society for Healthcare Epidemiology of America. Clin. Infect. Dis..

[38] Davey P., Brown E., Charani E., Fenelon L., Gould I.M., Holmes A., Ramsay C.R., Wiffen P.J., Wilcox M., Davey P. (2005). Interventions to improve antibiotic prescribing practices for hospital inpatients. Cochrane Database of Systematic Reviews.

[39] Ostrowsky B., Banerjee R., A Bonomo R., E Cosgrove S., Davidson L., Doron S., Gilbert D.N., Jezek A., Lynch J.B., Septimus E.J. (2018). Infectious Diseases Physicians: Leading the Way in Antimicrobial Stewardship. Clin. Infect. Dis..

[40] Price L., Gozdzielewska L., Young M., Smith F., MacDonald J., McParland J., Williams L., Langdridge D., Davis M., Flowers P. (2018). Effectiveness of interventions to improve the public’s antimicrobial resistance awareness and behaviours associated with prudent use of antimicrobials: A systematic review. J. Antimicrob. Chemother..

[41] Burstein V.R., Trajano R.P., Kravitz R.L., Bell R.A., Vora D., May L.S. (2019). Communication interventions to promote the public’s awareness of antibiotics: A systematic review. BMC Public Health.

[42] Gualano M.R., Gili R., Scaioli G., Bert F., Siliquini R. (2015). General population’s knowledge and attitudes about antibiotics: A systematic review and meta-analysis. Pharmacoepidemiol. Drug Saf..

[43] Dar O.A., Hasan R., Schlundt J., Harbarth S., Caleo G., Dar F.K., Littmann J., Rweyemamu M., Buckley E.J., Shahid M. (2016). Exploring the evidence base for national and regional policy interventions to combat resistance. Lancet.

[44] McLeod M., Ahmad R., Shebl N.A., Micallef C., Sim F., Holmes A. (2019). A whole-health–economy approach to antimicrobial stewardship: Analysis of current models and future direction. PLoS Med..

