# Supplementary material for: Knowledge, Attitudes, and Practices Regarding Antibiotic Use and Resistance: A Cross-Sectional Study from Oman
Source: Int J Environ Res Public Health. 2025 Nov 24;22(12):1778. doi: 10.3390/ijerph22121778 (PMC12732846; doi:10.3390/ijerph22121778)
Supplement: Supplementary file 1 [file ijerph-22-01778-s001.zip › ijerph-3833417-supplementary.pdf]

S1: Knowledge, Attitudes, and Practices Regarding Antibiotic Use and Resistance: A Cross-sectional Study from Oman

---

Demographic Information

1. **Age:** _____ years
2. **Gender:**
   - o Male
   - o Female
   - o Prefer not to say
3. **Nationality:**
   - o Omani
   - o Non-Omani (specify: _______)
4. **Education Level:**
   - o High school or below
   - o Diploma/Certificate
   - o Bachelor's degree
   - o Master's degree
   - o PhD/Doctorate
5. **Occupation:**
   - o Healthcare Professional (specify: Doctor/Nurse/Pharmacist/Other: _____)
   - o Non-healthcare professional
6. **Years of experience (for healthcare professionals):**
   - o Less than 5 years
   - o 5-10 years
   - o 11-20 years
   - o More than 20 years
7. **Work setting (for healthcare professionals):**
   - o Public hospital
   - o Private hospital
   - o Primary healthcare center
   - o Pharmacy

- Other: _____

DOMAIN 1: KNOWLEDGE ABOUT ANTIBIOTICS (20 Questions)

Section A: Basic Antibiotic Knowledge

8. **Antibiotics are effective against:**
    - Bacteria only
    - Viruses only
    - Both bacteria and viruses
    - Fungi only
    - Don't know
9. **Antibiotic resistance occurs when:**
    - Bacteria become resistant to antibiotics
    - Humans become allergic to antibiotics
    - Antibiotics lose their effectiveness over time
    - Doctors prescribe wrong antibiotics
    - Don't know
10. **Which of the following conditions should NOT be treated with antibiotics?**
    - Pneumonia
    - Common cold
    - Urinary tract infection
    - Skin infection
    - Don't know
11. **Taking antibiotics for viral infections:**
    - Helps recovery faster
    - Has no effect
    - Can contribute to antibiotic resistance
    - Prevents secondary bacterial infections
    - Don't know
12. **Broad-spectrum antibiotics:**
    - Are always better than narrow-spectrum antibiotics
    - Target a wide range of bacteria

- Should be used as first-line treatment
- Are less likely to cause resistance
- Don't know

Section B: Antibiotic Resistance Knowledge

13. **Antibiotic resistance is:**
    - A local problem only
    - A global health threat
    - Only a problem in developing countries
    - Not a serious concern
    - Don't know
14. **Which factor contributes MOST to antibiotic resistance?**
    - Overuse and misuse of antibiotics
    - Poor hygiene practices
    - Genetic factors
    - Environmental pollution
    - Don't know
15. **Stopping antibiotics early when feeling better:**
    - Is acceptable to avoid side effects
    - Can contribute to antibiotic resistance
    - Saves money
    - Has no impact on treatment
    - Don't know
16. **Sharing leftover antibiotics with family members:**
    - Is safe if they have similar symptoms
    - Should only be done with same household members
    - Can be harmful and contribute to resistance
    - Is acceptable to save healthcare costs
    - Don't know
17. **Agricultural use of antibiotics:**
    - Has no impact on human health
    - Can contribute to resistance in humans

- Is completely safe
- Only affects animals
- Don't know

Section C: Appropriate Use Knowledge

18. **The correct way to take antibiotics is:**
    - Take them until symptoms improve
    - Take the full prescribed course
    - Take extra doses for faster recovery
    - Skip doses if feeling better
    - Don't know
19. **Before prescribing antibiotics, healthcare providers should:**
    - Always do bacterial culture tests
    - Consider if infection is bacterial or viral
    - Prescribe the strongest antibiotic available
    - Always give broad-spectrum antibiotics
    - Don't know
20. **Self-medication with antibiotics:**
    - Is safe for minor infections
    - Should be avoided completely
    - Is acceptable if you've had the infection before
    - Is safe if using leftover prescribed antibiotics
    - Don't know

Section D: Advanced Knowledge

21. **Antimicrobial stewardship programs aim to:**
    - Reduce healthcare costs only
    - Optimize antibiotic use and reduce resistance
    - Eliminate all antibiotic use
    - Increase antibiotic availability
    - Don't know
22. **Infection prevention measures include:**
    - Hand hygiene only

- Hand hygiene, vaccination, and safe practices
- Antibiotic prophylaxis for everyone
- Isolation of all patients
- Don't know

23. **MRSA stands for:**
    - Methicillin-Resistant Staphylococcus Aureus
    - Multiple-Resistant Streptococcus Aureus
    - Methicillin-Related Skin Allergy
    - Multiple-Resistant Skin Allergy
    - Don't know

Section E: Local Context Knowledge

24. **In Oman, antibiotics can be obtained:**
    - Only with a prescription
    - Over-the-counter at pharmacies
    - Both with and without prescription
    - Only in hospitals
    - Don't know
25. **The most common cause of antibiotic resistance in Oman is:**
    - Hospital-acquired infections
    - Community overuse
    - Agricultural use
    - Imported resistant bacteria
    - Don't know
26. **Antibiotic resistance in Oman is:**
    - Not a significant problem
    - A growing concern
    - Completely under control
    - Unknown status
    - Don't know
27. **WHO recommendations for antibiotic use should be:**
    - Followed globally including Oman

- Adapted to local contexts only
- Ignored in developing countries
- Applied only in hospitals
- Don't know

DOMAIN 2: ATTITUDES AND BELIEFS (15 Questions)

Instructions: Please indicate your level of agreement with each statement using the scale: 1 = Strongly Disagree, 2 = Disagree, 3 = Neutral, 4 = Agree, 5 = Strongly Agree

28. **It is important to complete the full course of antibiotics even if feeling better.**
    - 1 (Strongly Disagree) - 2 (Disagree) - 3 (Neutral) - 4 (Agree) - 5 (Strongly Agree)
29. **Patients have the right to demand antibiotics from their doctors.**
    - 1 (Strongly Disagree) - 2 (Disagree) - 3 (Neutral) - 4 (Agree) - 5 (Strongly Agree)
30. **Antibiotic resistance is a serious global health threat.**
    - 1 (Strongly Disagree) - 2 (Disagree) - 3 (Neutral) - 4 (Agree) - 5 (Strongly Agree)
31. **It is acceptable to share antibiotics with family members with similar symptoms.**
    - 1 (Strongly Disagree) - 2 (Disagree) - 3 (Neutral) - 4 (Agree) - 5 (Strongly Agree)
32. **Healthcare professionals should educate patients about appropriate antibiotic use.**
    - 1 (Strongly Disagree) - 2 (Disagree) - 3 (Neutral) - 4 (Agree) - 5 (Strongly Agree)
33. **Public education campaigns about antibiotic resistance are necessary.**
    - 1 (Strongly Disagree) - 2 (Disagree) - 3 (Neutral) - 4 (Agree) - 5 (Strongly Agree)
34. **It is safe to keep leftover antibiotics for future use.**
    - 1 (Strongly Disagree) - 2 (Disagree) - 3 (Neutral) - 4 (Agree) - 5 (Strongly Agree)
35. **Doctors should prescribe antibiotics when patients expect them.**
    - 1 (Strongly Disagree) - 2 (Disagree) - 3 (Neutral) - 4 (Agree) - 5 (Strongly Agree)
36. **Stronger antibiotics are always better than weaker ones.**
    - 1 (Strongly Disagree) - 2 (Disagree) - 3 (Neutral) - 4 (Agree) - 5 (Strongly Agree)
37. **Antibiotic resistance affects everyone, not just those who misuse antibiotics.**
    - 1 (Strongly Disagree) - 2 (Disagree) - 3 (Neutral) - 4 (Agree) - 5 (Strongly Agree)
38. **Government regulations should control antibiotic sales in pharmacies.**
    - 1 (Strongly Disagree) - 2 (Disagree) - 3 (Neutral) - 4 (Agree) - 5 (Strongly Agree)
39. **Healthcare professionals need more training about antibiotic resistance.**

- 1 (Strongly Disagree) - 2 (Disagree) - 3 (Neutral) - 4 (Agree) - 5 (Strongly Agree)

40. **Agricultural use of antibiotics should be strictly regulated.**
    - 1 (Strongly Disagree) - 2 (Disagree) - 3 (Neutral) - 4 (Agree) - 5 (Strongly Agree)
41. **Individual actions can make a difference in fighting antibiotic resistance.**
    - 1 (Strongly Disagree) - 2 (Disagree) - 3 (Neutral) - 4 (Agree) - 5 (Strongly Agree)
42. **Fear of complications influences antibiotic prescribing decisions.**
    - 1 (Strongly Disagree) - 2 (Disagree) - 3 (Neutral) - 4 (Agree) - 5 (Strongly Agree)

DOMAIN 3: PRACTICES AND EXPERIENCES (10 Questions)

For All Participants:

43. **Have you ever taken antibiotics in the past 12 months?**
    - Yes
    - No
    - Don't remember
44. **How did you obtain your last course of antibiotics?**
    - Prescribed by a doctor
    - Bought from pharmacy without prescription
    - Used leftover antibiotics
    - Given by family/friends
    - Other: _____
45. **Have you ever stopped taking antibiotics before completing the full course?**
    - Never
    - Rarely
    - Sometimes
    - Often
    - Always
46. **Have you ever shared your antibiotics with others?**
    - Never
    - Rarely
    - Sometimes
    - Often

- Always

47. **Have you ever saved leftover antibiotics for future use?**
    - Never
    - Rarely
    - Sometimes
    - Often
    - Always
48. **Have you ever experienced antibiotic treatment failure?**
    - Yes
    - No
    - Not sure
49. **How often do you discuss antibiotic resistance with others?**
    - Never
    - Rarely
    - Sometimes
    - Often
    - Always
50. **Do you participate in awareness campaigns about antibiotic resistance?**
    - Yes, actively
    - Yes, occasionally
    - No, but interested
    - No, not interested

For Healthcare Professionals Only:

51. **How often do you consider antibiotic resistance patterns when prescribing?**
    - Never
    - Rarely
    - Sometimes
    - Often
    - Always
52. **What influences your antibiotic prescribing decisions most?** (Select all that apply)
    - Clinical guidelines

- Patient expectations
- Time constraints
- Diagnostic uncertainty
- Fear of complications
- Resistance patterns
- Cost considerations

Open-ended Questions:

53. **What do you think are the main causes of antibiotic resistance in Oman?**

---

54. **What strategies do you suggest to reduce antibiotic resistance in Oman?**

---

55. **Any additional comments about antibiotic use and resistance:**

Thank you for participating in this important study. Your responses will help improve antibiotic use and combat resistance in Oman.
